# A Síndrome de Takotsubo Existe como uma Doença Específica?

**DOI:** 10.36660/abc.20200470

**Published:** 2020-08-19

**Authors:** Bruno Caramelli

**Affiliations:** 1 Universidade de São Paulo Instituto do Coração São Paulo SP Brasil Universidade de São Paulo – Instituto do Coração (InCor),São Paulo, SP – Brasil

**Keywords:** Cardiomiopatia de Takotsubo, Disfunção Ventricular, Doença Arterial Coronariana, Diagnóstico Diferencial, Diagnóstico por Imagem

O nome “síndrome” combina duas raízes gregas para descrever uma condição que reúne um grupo de sinais e sintomas que existem juntos nos pacientes. Parece que Avicenna o utilizou pela primeira vez em sua publicação de 1025, “ *The Canon of Medicine* ”.^1^ Em genética, o uso da palavra “síndrome” geralmente pressupõe que a causa subjacente da doença seja conhecida. Por outro lado, em medicina, a síndrome refere-se a condições de causa conhecida e desconhecida.

Historicamente, os sinais e sintomas associados considerados de correlação improvável acabaram sendo conhecidos por terem uma causa subjacente responsável por todos eles. Mesmo depois que a causa é revelada, a palavra original permanece, às vezes com o nome do primeiro descritor, e essa é provavelmente a razão da existência hoje em dia de síndromes com causas identificadas e não identificadas.

Nesta edição, os “Arquivos Brasileiros de Cardiologia” publicam o artigo intitulado “Registro multicêntrico de Takotsubo (REMUTA) – Aspectos clínicos,

Desfechos intra-hospitalares e mortalidade em longo prazo.”^2^ A síndrome de Takotsubo foi relatada inicialmente por Sato et al., em 1990, no Japão, descrevendo 16 casos que compartilhavam sinais e sintomas conhecidos: dor torácica típica após um evento estressante e artérias coronárias “angiograficamente normais”.

O Registro Multicêntrico de Takotsubo (REMUTA) adotou os critérios de diagnóstico da força-tarefa sobre a síndrome de Takotsubo da “ *Heart Failure Association of the European Society of Cardiology* ”, publicada em 2016.^3^ De acordo com esses critérios, se a lesão coronariana culpada for identificada, o diagnóstico de síndrome coronariana aguda é estabelecido e a síndrome de Takotsubo é descartada. No registro REMUTA, todos os pacientes foram submetidos à angiografia coronariana e 24,2% deles apresentaram doença arterial coronariana não-obstrutiva, definida pelos autores como menos de 50% de obstruções. Os 75,8% restantes tinham, segundo os autores, artérias coronárias “angiograficamente normais”. Entretanto, não há referência à ausência de lesão coronariana culpada nos pacientes estudados, critério necessário para o diagnóstico da síndrome de Takotsubo, conforme declarado no primeiro consenso de 2016, adotado pelo estudo REMUTA.

Acima, a expressão “angiograficamente normal” está entre aspas de propósito, por um motivo: a definição de artérias coronárias angiograficamente normais é um desafio para ser estabelecido. Existem limitações relacionadas à resolução dos métodos de imagem e ao conceito bem conhecido de que mesmo as placas ateroscleróticas sutis podem desencadear trombose coronariana. Além disso, a disfunção endotelial que afeta as artérias coronárias epicárdicas ou suas ramificações endocárdicas pode ser responsável pelo desenvolvimento de síndromes coronarianas agudas (SCA). De fato, um consenso internacional sobre imagens intracoronarianas, publicado em 2019, afirma que a lesão culpada não pode ser identificada em 4-10% dos pacientes com SCA com supradesnivelamento do segmento ST e em >30% dos pacientes sem supradesnivelamento do segmento ST.^4^ Considerando essas advertências, mesmo após modificações, a definição angiográfica da síndrome de Takotsubo não oferece um critério à beira do leito seguro para a prática clínica. Talvez por esse motivo, em 2018, a definição original da síndrome de Takotsubo, sugerida por Sato et al. foi modificada novamente, e o novo consenso afirmou que doença arterial coronariana significativa não é mais uma contradição para o diagnóstico da síndrome de Takotsubo.^5^

Na Medicina, é de suma importância ter condições de saúde com definições claras para o diagnóstico diferencial, estratificação de risco e como referência para estudos futuros em pesquisas clínicas. Esse princípio abriu o caminho para o estabelecimento dos conceitos agora centrais de síndromes coronarianas agudas com supradesnivelamento e sem supradesnivelamento do segmento ST, por exemplo. À beira do leito, os cardiologistas modernos sabem como estratificar o risco e dar tratamento adequado para ambas as condições. Este não é o caso da síndrome de Takotsubo, que não pode ser indiscutivelmente diferenciada das síndromes coronárias agudas. Como pode ser observado na Tabela 1 , exceto pelo aumento da prevalência em mulheres na pós-menopausa, todos os outros critérios estão presentes nas duas síndromes e as diferenças são baseadas em opiniões subjetivas tomadas na frente do paciente. Na ausência de critérios objetivos, o diagnóstico diferencial é desafiador e os pacientes podem ser diagnosticados erroneamente. Apesar de considerar muito importante ter um Registro Nacional de uma doença específica (e parabenizo os autores por esse esforço), o leitor não pode excluir a possibilidade de alguns pacientes no registro REMUTA terem o diagnóstico de SCA em vez de Síndrome de Takotsubo.

**Tabela 1:** – Gráfico de comparação de critérios diagnósticos entre a síndrome de Takotsubo e síndrome coronariana aguda

Critérios diagnósticos	Takotsubo	Síndrome coronariana aguda
Disfunção transitória do ventrículo esquerdo	+++	+
Gatilho emocional / físico	++	+
Distúrbios neurológicos como gatilho	++	+
Novas anormalidades no ECG	++	+++
Biomarcadores cardíacos elevados	+	+++
Descartar miocardite por infecção	+	+
Mais frequente em mulheres na pós-menopausa	+	-
Doença arterial coronariana significativa	+	+++

Em resumo, o diagnóstico correto da síndrome de Takotsubo muitas vezes permanece indefinido. Por outro lado, dados recentes mostraram que a ressonância magnética tem um papel promissor na síndrome de Takotsubo e pode representar, no futuro, a base para o diagnóstico diferencial.^6^ As síndromes coronarianas agudas representam hoje um grande e importante grupo de doenças cardíacas, cada uma com características e tratamento específicos. A síndrome de Takotsubo, pelo contrário, ainda carece de uma identidade definitiva. Ela é uma doença específica e independente ou uma apresentação peculiar de uma síndrome coronariana aguda? Como na versão hebraica da história de Jonas na Bíblia, a Takotsubo deve nadar mais rápido, ficar mais forte e crescer; caso contrário, será engolida pelo peixe gigante.

**Figura 1:**
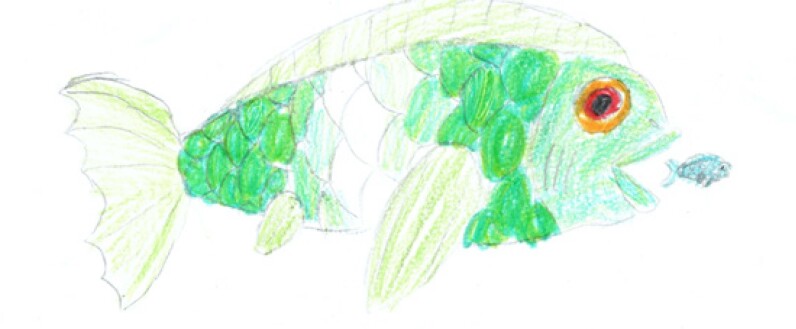
– *Como na versão hebraica da história de Jonas na Bíblia, a Takotsubo deve nadar mais rápido, ficar mais forte e crescer; caso contrário, será engolida pelo peixe gigante. Arte de Piero de Souza Dias Caramellli.*
