# Supplementary material for: A Síndrome de Takotsubo Existe como uma Doença Específica?
Source: Arq Bras Cardiol. 2020 Aug 19;115(2):217–8. [Article in Portuguese] doi: 10.36660/abc.20200470 (PMC8384284; doi:10.36660/abc.20200470)
Supplement: Translation

# Translation: Takotsubo Syndrome, Does it Exist as a Specific Disease?

**Authors:** Bruno Caramelli

**Affiliations:** 1 Brazil Universidade de São Paulo – Instituto do Coração (InCor), São Paulo, SP – Brazil

**Keywords:** Takotsubo Cardiomyopathy, Ventricular Dysfunction, Coronary Artery Disease, Diagnosis,Differencial, Diagnostic,Imaging

The name syndrome combines two Greek roots to describe a condition gathering a group of signs and symptoms existing together in patients. It seems that Avicenna first used it in his 1025 publication “The Canon of Medicine”.^1^ In genetics, the use of the name syndrome generally assumes that the underlying cause of the disease is known. On the other hand, in medicine, syndrome refers to conditions both with a known and unknown cause.

Historically, associated signs and symptoms found to be of improbable correlation were eventually known to have an underlying cause responsible for all of them. Even after the cause is unraveled, the original word remains, sometimes with the name of the first describer, and this is probably the reason for the existence nowadays of syndromes with identified and unidentified causes.

In this number, *Arquivos Brasileiros de Cardiologia* publishes the article entitled Takotsubo Multicenter Registry (REMUTA) - Clinical aspects, in-hospital outcomes, and long-term mortality.^2^ The Takotsubo syndrome was reported initially by Sato et al., in 1990, in Japan, describing 16 cases that shared well-known signs and symptoms: typical chest pain following a stressful event and “angiographically normal” coronary arteries.

The Takotsubo Multicenter Registry (REMUTA) adopted the diagnostic criteria from the task force on Takotsubo syndrome of the Heart Failure Association of the European Society of Cardiology, published in 2016.^3^ According to these criteria, if the culprit coronary lesion is identified, the diagnosis of acute coronary syndrome is established, and Takotsubo syndrome is discarded. In the REMUTA registry, all patients performed coronary angiography, and 24.2% of them showed non-obstructive coronary artery disease, defined by the authors as less than 50% obstructions. The remaining 75.8% had, according to the authors, “angiographically normal” coronary arteries. However, there is no reference to the absence of a culprit coronary lesion in the patients studied, a necessary criterion for the diagnosis of Takotsubo syndrome, as stated in the first 2016 consensus, adopted by the REMUTA study.

Above, the expression “angiographically normal” has quotes, on purpose, for one reason: the definition of angiographically normal coronary arteries is challenging to establish. There are limitations related to the resolution of imaging methods and the well-known concept that even subtle atherosclerotic plaques can trigger coronary thrombosis. Moreover, endothelial dysfunction affecting epicardial coronary arteries or its endocardial ramifications can be responsible for the development of acute coronary syndromes (ACS). Indeed, an International consensus about intracoronary imaging, published in 2019, states that the culprit lesion cannot be identified in 4–10% of the patients with ST-elevation ACS and in >30% of patients non-ST-elevation.^4^ Considering all these caveats, even after modifications, the angiographic definition for Takotsubo syndrome does not offer a confident bedside criterion for clinical practice. Maybe for that reason, in 2018, the original definition of Takotsubo syndrome, suggested by Sato et al. was modified again, and the new consensus stated that significant coronary artery disease is not anymore, a contradiction for the diagnosis of Takotsubo syndrome.^5^

In medicine, it is of paramount importance to have health conditions with clear definitions for differential diagnosis, risk stratification, and as reference for future studies in clinical research. This principle paved the way for the establishment of the now central concepts of ST-elevation and non-ST-elevation acute coronary syndromes, for example. At the bedside, modern cardiologists know how to stratify risk and give adequate treatment for both conditions. This is not the case for Takotsubo syndrome that cannot be undoubtedly distinguishable from acute coronary syndromes. As can be seen in Table 1 , except for the increased prevalence in postmenopausal women, all other criteria are present in both syndromes, and the differences are based on subjective opinions taken in front of the patient. In the absence of objective criteria, differential diagnosis is challenging, and the patients could be misdiagnosed. Despite considering very important to have a National Registry of a specific disease (and I congratulate the authors for that effort), the reader cannot exclude the possibility that some patients in REMUTA had the diagnosis of ACS instead of Takotsubo Syndrome.

**Table 1:** – Diagnostic criteria comparison chart between Takotsubo and Acute coronary syndromes

Diagnostic criteria	Takotsubo	Acute coronary syndromes
Transient left ventricular dysfunction	+++	+
Emotional/physical trigger	++	+
Neurological disorders as a trigger	++	+
New ECG abnormalities	++	+++
Elevated cardiac biomarkers	+	+++
Discard infection myocarditis	+	+
More frequent in postmenopausal women	+	-
Significant coronary artery disease	+	+++

In summary, the correct diagnosis of Taktsubo syndrome often remains elusive. Conversely, recent data showed that magnetic resonance has a promising role in Takotsubo syndrome and could represent, in the future, the cornerstone for the differential diagnosis.^6^ Acute coronary syndromes represent today a big and important group of heart diseases, each one with specific characteristics and treatment. Takotsubo syndrome, on the contrary, still lacks a definitive identity. Is it a specific, independent disease or a peculiar presentation of an acute coronary syndrome? As in the Hebrew version of the story of Jonah in the Bible, Takotsubo must swim faster, get stronger and grow; otherwise, it will be swallowed by the giant fish.

**Figure 1:** – As in the Hebrew version of the story of Jonah in the Bible, Takotsubo must swim faster, get stronger and grow; otherwise, it will be swallowed by the giant fish. Art by Piero de Souza Dias Caramellli.
